# Antidepressant Effect of* Fraxinus rhynchophylla* Hance Extract in a Mouse Model of Chronic Stress-Induced Depression

**DOI:** 10.1155/2018/8249563

**Published:** 2018-07-03

**Authors:** Yu Ri Kim, Bo-Kyung Park, Young Hwa Kim, Insop Shim, In-Cheol Kang, Mi Young Lee

**Affiliations:** ^1^Clinical Medicine Division, Korea Institute of Oriental Medicine, Daejeon 34054, Republic of Korea; ^2^Department of Physiology, School of Medicine, Kyung Hee University, 1 Kyungheedae-ro, Dongdaemun-gu, Seoul 02454, Republic of Korea; ^3^Department of Biological Science, College of Life and Health Sciences, Hoseo University, Asan 31499, Republic of Korea

## Abstract

Prolonged exposure to stress can affect mood and cognition and lead to mood disorders. Research on stress-associated mood disorders is important in modern society as people are increasingly exposed to unavoidable stressors. We used a mouse model with 2 weeks of exposure to electric foot shock and restraint, to determine the effect of* Fraxinus rhynchophylla* Hance (FX) extract on chronic stress-induced depression. We measured the effect of FX extract using various physiological, behavioral, and biochemical measures. FX extract ameliorated chronic stress-induced body and relative liver weight loss and improved depressive-like behaviors in the open field and forced swim tests. In addition, plasma cortisol and serotonin levels in stress-induced mice following FX treatment were similar to normal mice, and the elevation of proinflammatory cytokines was prevented. Moreover, FX treatment increased the expression of phosphorylated cyclic adenosine-3′,5′-monophosphate response element-binding protein (pCREB)/brain-derived neurotrophic factor (BDNF). Further experiments confirmed the efficacy of FX extract by showing similar results using esculin and esculetin, compounds extracted from FX. Taken together, these results indicate that FX extract has an antidepressant effect on chronic stress-induced depression by associating signaling with neuroinflammation and neurogenesis.

## 1. Introduction

Major depression is the most common mood disorder, with a lifetime prevalence of less than 20% and high recurrence rate of 65% [1–3]. Depression leads to changes in mood and cognitive function and can affect the personal relationships and social life of those affected [4, 5]. The exact etiology of depression is unknown; however, depressive disorders may be caused by many factors, including life events, personality, gender identity and sexuality, medical treatments, nonpsychiatric illnesses, and psychiatric syndromes [6–9].

Stress is a major risk factor for depression and chronic unpredictable stress can impair brain function, behavior and cognition, digestion, and endocrine function [10–13]. Models of chronic unpredictable stress have been designed to mimic human depression and posttraumatic stress disorder in rodents [14–16]. These models have shown that chronic unpredictable stress reduces the sensitivity of serotonin neurotransmission and its receptors in the medial prefrontal cortex, amygdala, hippocampus, and dorsal raphe. These decreases result in depressive-like phenotypes, such as anhedonia and despair, and reduce hippocampal neurogenesis [16, 17]. Prolonged psychological stress also promotes cortisol secretion, which reduces synaptic density and neuronal survival in the prefrontal cortex and hippocampus [18].

Chronic mild stress results in an increase in the expression of* interleukin (IL)-1β* mRNA and protein in the hippocampus, serum, and cerebrospinal fluid of rats* via* the nuclear factor kappa-light-chain-enhancer of activated B cells (NF-*κ*B) inflammatory pathway [19, 20]. In addition, IL-6, IL-12, and tumor necrosis factor-*α* (TNF-*α*) are increased in the serum of rats exposed to chronic stress [21, 22]. These inflammatory cytokines are associated with the expression of stress hormones [23].

Antidepressants were created based on the monoamine hypothesis that depression is caused by an imbalance in the monoamine neurotransmitters. There are different classes of neurotransmitters, including selective serotonin reuptake inhibitors (SSRIs), norepinephrine reuptake inhibitors (NRIs), tricyclic antidepressants (TCAs), and monoamine oxidase inhibitors (MOIs) [24–26]. Side effects, such as sexual dysfunction, coronary heart disease, and fracture risk, have been reported with the use of these antidepressants [27–29]; therefore, Traditional Chinese Medicine (TCM), which reports significantly fewer side effects than modern medicine, is being researched as an alternative to modern antidepressants. Natural herbs are attracting attention as therapeutic agents that can be administered over long periods with a reduced cost burden [30].* Fraxinus rhynchophylla* Hance (FX) belongs to the Oleaceae family and is mainly distributed in Korea and China. The stem bark of FX (*Fraxini cortex*, Qinpi) is described in Donguibogam, the foremost historical text on Traditional Korean Medicine [31]. It is included in the “heat-clearing" category, where treatments for diarrhea, germs, arthritis, and hyperuricemia are found [32]. However, the antidepressant effect and mechanism of action of FX extract on chronic stress have not been elucidated.

We sought to evaluate the potential of FX extract as medicine by preventing depressive-like behavior after chronic stress. We hypothesized that FX extract may have an antidepressant-like effect on depression caused by two weeks of electric foot shock and restraint in mice.

## 2. Materials and Methods

### 2.1. Animals with Treatments

**Figure 1:**
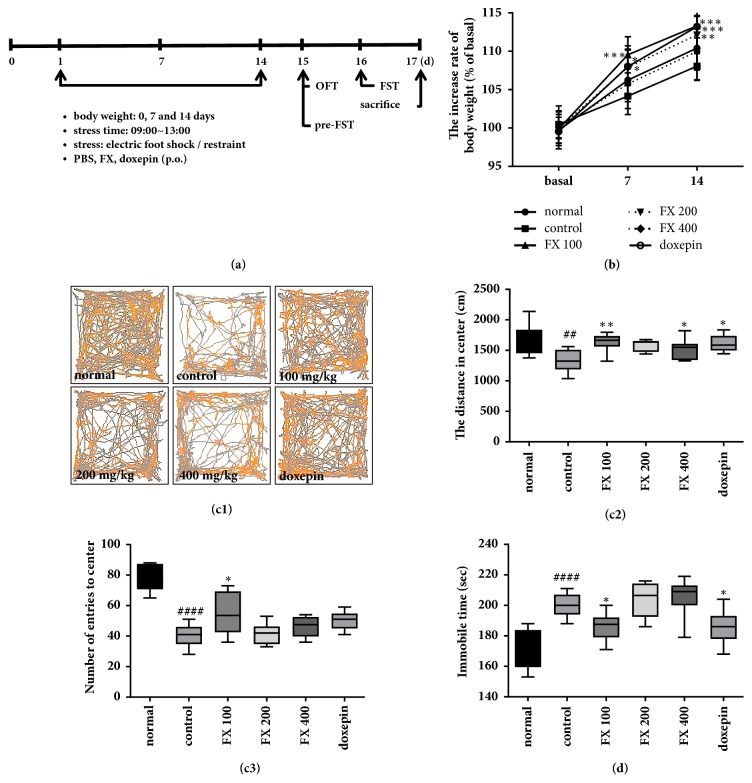
**Effect of FX extract on body weight and depressive-like behavior**. (a) Experimental procedure schematic. (b) Body weight in all mice groups as a % of baseline body weight (n = 8-10). (c1) Representative recordings of total distance travelled in Open Field Test in all groups. (c2) Differences between the total distances travelled in center (n = 8-9). (c3) Differences between mean numbers of entries into center in all groups (n = 8). (d) Mean immobility time between groups in the Forced Swim Test (n = 11). Mean ± SD. ^##^*P* < 0.01 and ^####^*P* < 0.0001 versus normal group; ^*∗*^*P* < 0.05, ^*∗∗*^*P* < 0.01, and ^*∗∗∗*^*P* < 0.001 versus control group.

Male c57BL/6 mice, aged 7 weeks, were obtained from DBK Co., Ltd. (Eumseong-gun, Chungcheongbuk-do, Republic of Korea). The mice were housed at 22–24°C under alternating 12 h dark/light cycles in specific-pathogen-free (SPF) conditions. They were fed a commercial diet and allowed tap water* ad libitum* throughout the study. After a week of habituation, mice were randomly divided into the following groups for the first experiment: non-stress + PBS (normal), stress + PBS (control), stress + FX 100 mg·kg^−1^ (FX 100), stress + FX 200 mg·kg^−1^ (FX 200), stress + FX 400 mg·kg^−1^ (FX 400), and stress + doxepin 15 mg·kg^−1^ (doxepin, positive control, Sigma-Aldrich, St. Louis, MO, USA). Groups for the second experiment were as follows: normal, control, stress + esculin 50 mg·kg^−1^ (esculin, Sigma-Aldrich), stress + esculetin 50 mg·kg^−1^ (esculetin, Sigma-Aldrich), and doxepin (Sigma-Aldrich). PBS, FX extracts, esculin, esculetin, and doxepin were administered orally. All experiments were approved by the Committee on Animal Care of KIOM (17-025 and 17-099) and Use Committee in accordance with the National Institutes of Health Guidelines. A schematic of the experimental timeline is shown in Figure 1(a).

### 2.2. Preparation of* F. rhynchophylla* Hance Extract

Ethanol stem bark extract of FX was prepared by sonicating dried ground powder (171.86 g) suspended in 70% ethanol solvent for 3 h. After filtration, the extract was lyophilized and powdered. The extract was processed as described for ethanol stem bark extraction, yielding 150.00 g (Yield 11.46 %) [33].

### 2.3. Components Analysis of* F. rhynchophylla* Hance Extract

10 mg of FX extract was dissolved in 70% ethanol and the solution was filtered with a 0.2 *μ*m polyvinylidene difluoride membrane (PVDF) filter to prepare an analytical sample. The analytical sample was analyzed using high-performance liquid chromatography-ultraviolet (HPLC-UV) analysis to determine the peak areas of the four main components of the dermis to compare to a previously prepared calibration curve to determine the concentration of each principal component in the extract solution. Using this, the mg·g^−1^ content of each main component in 70% ethanol extract of dermis was determined. Each analytical sample was analyzed in triplicate.

### 2.4. Chronic Stress

With the exception of the normal group, all mice were exposed to chronic stress daily between 09:00 and 13:00 for 2 weeks. After one hour of administration, mice received an electric foot shock (intensity, 0.5 mA) for 1 s, with an intershock interval of 10 s, for total 2 min in the electronic shock generator (JD-SI-10, JEUNG DO BIO & PLANT CO., Ltd., Nowon-gu, Seoul, Republic of Korea). This generator is 410 × 210 × 300 mm with stainless steel rods connected to a shock generator. Following this, mice were locked in a cylindrical acrylic restrainer (ø30 × 100 mm; JD-R-05A, JEUNG DO BIO & PLANT CO., Ltd.) for total 2 h.

### 2.5. Body Weight and Liver Index

To determine changes in body weight, we measured body weight prior to the chronic stress period (basal) and at days 7 and 14 during chronic stress. The basal measurement was converted to 100% and changes at days 7 and 14 were expressed as a percentage. Liver weight was measured postmortem. The liver index was calculated as a percentage of liver/body weight. This calculation is based on the study that the liver weight increases relative to the body weight in animals exposed to restraint stress [34].

### 2.6. Behavioral Tests

The open field test (OFT) and forced swim test (FST) were performed to assess depressive-like behaviors for 2 days following chronic stress (days 15-16).

#### 2.6.1. Open Field Test

The OFT measures levels of anxiety in rodents exposing them to a new environment and measuring their activity. The process of this experiment was based on Liu et al., 2014 [35]. This test was performed in white acrylic box (30 × 30 × 40 cm) using Ethovision XT 9 (Noldus Information Technology, Wageningen, The Netherlands). The distance travelled in arbitrarily designated central zone (10 × 10 cm) of the box and number of entries into this zone were recorded for 10 min.

#### 2.6.2. Forced Swim Test

The FST measures the degree of behavioral despair in an inescapable space. The process of this experiment was based on Can et al., 2012 [36]. The day before the test, the mice were exposed to a transparent acrylic cylinder (H: 60 cm, D: 16 cm) filled with water at 25°C for 15 min. On the day of the test, mice were recorded in the water for total 6 min using Smart3.0 (Panlab, S.L.U, Barcelona, Spain). Depressive-like behavior was assessed by measuring immobility time for 4 min of the recording after the first 2 min of latency time.

### 2.7. Enzyme-Linked Immune-Specific Assay (ELISA)

On day 17, mice were anesthetized with zoletil (1 mg·kg^−1^; i.p.) and cardiac puncture was performed to collect blood. Blood was centrifuged at 1000 g for 10 min. The upper fluid (plasma) was stored at –70°C. The concentration of serotonin, a neurotransmitter known to be reduced in depressive disorders, and cortisol, a glucocorticoid hormone secreted from the adrenal cortex in stressful conditions, in plasma was analyzed using Serotonin ELISA Kit (Abcam, Cambridge, UK) and Cortisol ELISA Kit (Cayman Chemical, Ann Arbor, Michigan, USA). Plasma samples, diluted 1/10, and protein standards were loaded on a 96-well plate. After reagents were added according to the manufacturer's instructions, the plate was read using a VersaMax microplate reader (Molecular Devices, Sunnyvale, CA, USA) and the absorbance was measured at the appropriate optical density using SoftMax pro 6.2.2 (Molecular Devices).

### 2.8. Western Immunoblotting

Brain tissue (prefrontal cortex and hippocampus) was homogenized in lysis buffer and equalized to the same total amount of protein. Samples were separated by 4–20% Mini-PROTEAN® TGX™ Precast Protein Gels (Bio-Rad Laboratories, Inc., Hercules, CA, USA) and separated proteins were transferred to a PVDF (Amersham Biosciences, Piscataway, NJ, USA). Membranes were incubated with primary antibody overnight at 4°C: actin (Sigma-Aldrich, St. Louis, MO, USA), brain-derived neurotrophic factor (BDNF; Cat. Ab108319, Abcam), cyclic adenosine-3′,5′-monophosphate response element-binding protein (CREB; Cat. 9197, Cell Signaling Technology, Danvers, MA, USA), phosphorylated CREB (pCREB; Cat. 9198, Cell Signaling Technology). Following this, membranes were incubated with appropriate secondary antibodies at room temperature (RT). Actin was used as a loading control for all experiments. The density of the protein band was quantified using an ImageQuant LAS 4000 mini (Fujifilm, Tokyo, Japan).

### 2.9. Real-Time Polymerase Chain Reaction (Real-Time PCR)

Brain tissue (prefrontal cortex and hippocampus) was homogenized using easy-BLUE™ reagent and the RNA pellet was extracted. The complementary deoxyribonucleic acid (cDNA) was synthesized by equalizing all samples to the same total amount of RNA and adding a synthetic reagent. Real-time PCR primers were loaded onto a MicroAmp Fast 96-well reaction plate (Applied Biosystems) with Power SYBR Green PCR Master Mix (Applied Biosystems) and mRNA was quantified using Quantstudio 6 Flex (Applied Biosystems by Life Technologies). The sequences of the real-time PCR primers were as follows: 5′-CTTGGCAACAGCACAGACC-3′ (forward), and 5′-GAGAAGTCCATGTCCGCAAT-3′ (reverse) for* NF-κB p65*; 5′- AGACCCTCACACTCAGAT CATCTTC-3′ (forward) and 5′-CCACTTGGTGGTTTGCTACGA-3′ (reverse) for* TNFα*; 5′-GAGGATACCACTCCCAACAGACC-3′ (forward) and 5′-AAGTGCATCATCGTTGTTCATACA-3′ (reverse) for* IL-6*; 5′-AGACATGGAGTCATAGGCTCTG-3′ (forward) and 5′-CCATTTTCCTTCTT GTG GAGCA-3′ (reverse) for* IL-12*; and 5′-AAGGTGGTGAAGCAGGCA T-3′ (forward) and 5′-GGTCCAGGGTTTCTTACTCCT-3′ (reverse) for* glyceraldehyde-3-phosphate dehydrogenase (GAPDH)*. Real-time PCR was performed by incubating the mixture and PCR thermal cycle parameters were as follows: 95°C for 10 min, 40 cycles of 60°C for 60 s, and 95°C for 15 s, and a melting curve from 60 to 95°C to ensure amplification of a single product.

### 2.10. Immunofluorescence

Mice were anesthetized with zoletil (1 mg·kg^−1^; i.p.) and perfused* via* cardiac puncture with PBS and 4% paraformaldehyde solution. Immunofluorescence staining was performed following the standard immunofluorescence protocol of Cell Signaling Technology. Brain sections (30 *µ*m) were fixed with 4% paraformaldehyde solution for 15 min and then blocked using blocking buffer (1× PBS/5% normal goat serum/0.3% Triton X-100) for 1 h. Primary antibodies (BDNF: Cat. NB100-98682, Novus Biologicals, LLC, Littleton CO, USA; pCREB: Cat. 9198, Cell Signaling Technology) were diluted 1:500 with antibody dilution buffer (1× PBS/1% BSA/0.3% Triton X-100) and incubated on the sample overnight at 4°C. Following this, slides were incubated with secondary antibodies (1:500) for 2 h at room temperature (RT). Slides were mounted with Fluoroshield Mounting Medium with 4′,6-diamidino-2-phenylindole (DAPI; Abcam). Images (20×) were collected using a fluorescence microscope (Nikon Instruments Inc., Tokyo, Japan), and integrated optical density (I.O.D.) was measured using an I-solution (IMT i-Solution Inc., Burnaby, BC, Canada).

### 2.11. Data Analysis

All data are expressed as mean ± standard deviation (SD) and analyzed using GraphPad Prism 7 (GraphPad Software, Inc., La Jolla, CA, USA). Statistical analysis was performed using one-way and repeated one-way analysis of variance (ANOVA) with Tukey's post hoc comparisons. P values < 0.05 were considered statistically significant.

## 3. Results

### 3.1. Behavioral Effect of* F. rhynchophylla* Hance Extract on Stress-Induced Depression

To verify the effect of FX extract on stress-induced depression, all groups of mice were assessed for their body weight, OFT, and FST. Mice were weighed before stress (basal), both at 7 and 14 days after chronic stress. There were no significant differences in measurements between normal and control groups. However, treatment with FX 100, FX 200, and doxepin groups showed a significant recovery in body weight (Figure 1(b); F_(5,143)_ = 6.493, p < 0.0001). Depressive-like behaviors were significantly higher in the control group when compared with the normal group (Figures 1(c) and 1(d)). The distance travelled in the center of the OFT was significantly lower in FX 100, FX 200, and doxepin groups when compared with the control group (Figure 1(c2); F_(5,46)_ = 4.408, p = 0.0023). In addition, the number of entries into the center was significantly increased in FX 100 group (Figure 1(c3); F_(5,42)_ = 20.4, p < 0.0001). Immobility time was significantly increased in the FST by treatment with 100 mg·kg^−1^ FX extract when compared with the control group (Figure 1(d); F_(5,61)_ = 19.68, p < 0.0001). We repeated the experiment with 30, 50, and 100 mg·kg^−1^ FX extract. No significant antidepressant effect was observed at 30 and 50 mg·kg^−1^ FX extract (data not shown). These results suggest that mice exposed to chronic stress developed markedly depressive-like behaviors and FX significantly improves these behaviors.

### 3.2. Effect of* F. rhynchophylla* Hance Extract on Cortisol and Serotonin Concentrations

**Figure 2:**
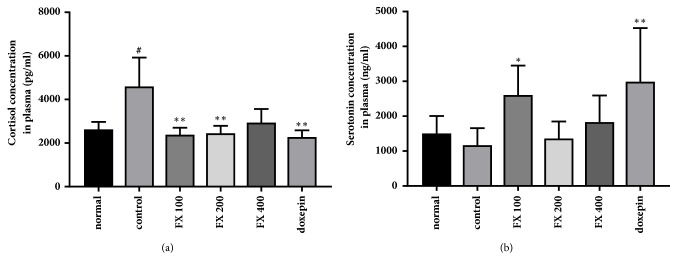
**Effect of FX extract on plasma cortisol and serotonin concentrations**. (a) The concentration of plasma cortisol in all groups (n = 8). (b) The concentration of plasma serotonin in all groups (n = 8). Mean ± SD. ^#^*P* < 0.05 versus normal group; ^*∗*^*P* < 0.05 and ^*∗∗*^*P* < 0.01 versus control group.

To determine whether treatment with FX extract affects the concentration of stress-related hormones and depression-related neurotransmitters, respectively, we measured changes in cortisol and serotonin concentration in the plasma of mice. The concentration of cortisol was significantly increased by chronic stress; however this increase was ameliorated by treatment with FX extract and doxepin (Figure 2(a); F_(5,42)_ = 4.395, p = 0.0026). The concentration of serotonin showed a tendency to decrease with chronic stress and was increased by treatment with 100 mg·kg^−1^ FX extract (Figure 2(b); F_(5,38)_ = 5.224, p = 0.001). These results suggest that 100 mg·kg^−1^ FX extract prevents chronic stress-induced changes in the concentration of plasma cortisol and serotonin.

### 3.3. Effect of* F. rhynchophylla* Hance Extract on the Expression of* NF-κB p65*,* IL-12*,* IL-6*, and* TNF-α* mRNA

**Figure 3:**
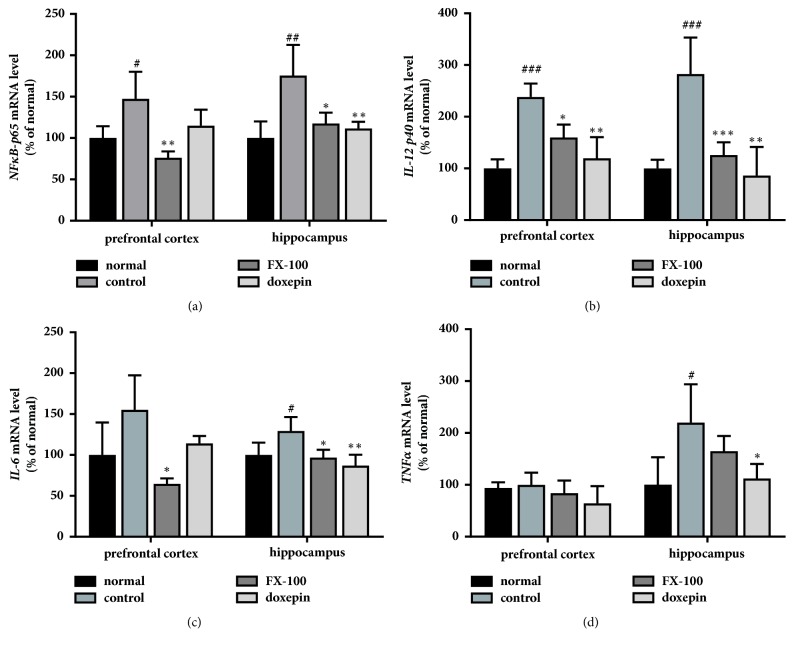
**Effect of FX extract on the expression of NF-**κ**B, TNF-**α**, IL-6, and IL-12 mRNA**. (a) The expression of* NF-κB p65* mRNA in the prefrontal cortex and hippocampus in all groups (n = 4). (b-d) The expression of the inflammatory cytokines,* IL-12, IL-6,* and* TNFα* mRNA in the prefrontal cortex and hippocampus (n = 3-4). Mean ± SD. ^#^*P* < 0.05, ^##^*P* < 0.01, and ^###^*P* < 0.001 versus normal group; ^*∗*^*P* < 0.05, ^*∗∗*^*P* < 0.01, and ^*∗∗∗*^*P* < 0.001 versus control group.

Prolonged exposure to stress increases the mRNA expression of proinflammatory cytokines,* IL-1β*,* IL-6*, and* TNF-α*, in the cortex and hippocampus [37, 38]. We assessed whether treatment with FX extract can alter cytokine mRNA expression using real-time PCR. The expression of* NF-κB p65* mRNA, a factor that induces the production of inflammatory cytokines, was significantly increased in the prefrontal cortex and hippocampus following chronic stress. This increase was attenuated by treatment with 100 mg·kg^−1^ FX extract or doxepin (Figure 3(a); P.C., F_(3,11)_ = 8.205, p = 0.0038; Hippo, F_(3,12)_ = 9.046, p = 0.0021). In addition, we found that expression of* IL-12* and* IL-6* mRNA was increased by chronic stress in the prefrontal cortex and hippocampus and this effect was significantly attenuated by treatment with 100 mg·kg^−1^ FX extract or doxepin (Figure 3(b); P.C., F_(3,11)_ = 14.96, p = 0.0003; Hippo, F_(3,12)_ = 14.68, p = 0.0003 and Figure 3(c); P.C., F_(3,11)_ = 4.791, p = 0.034; Hippo, F_(3,13)_ = 7.229, p = 0.0042). The expression of* TNF-α* mRNA is increased by chronic stress in the hippocampus, which is significantly decreased by treatment with doxepin but not FX extract (Figure 3(d); Hippo, F_(3,17)_ = 11.59, p = 0.0002). These results suggest that FX extract modulates the expression of* IL-12* and* IL-6* mRNA in the prefrontal cortex and hippocampus, which is mediated by NF-*κ*B p65, in chronic stress-induced depression.

### 3.4. Effect of* F. rhynchophylla* Hance Extract on CREB/BDNF Signaling

**Figure 4:**
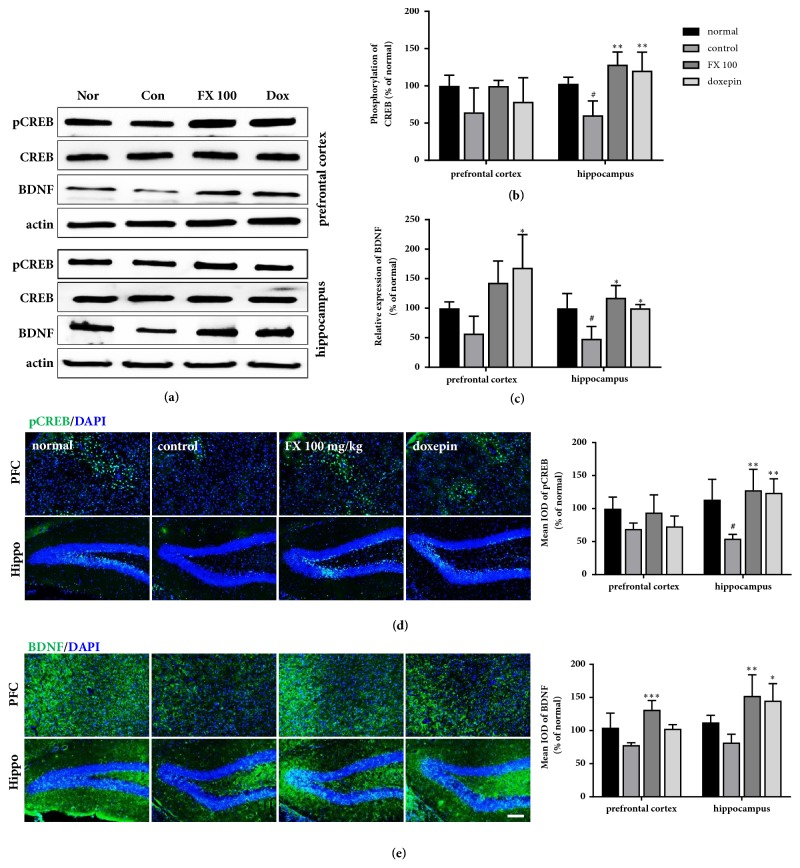
**Effect of treatment with FX extract on CREB/BDNF signaling**. (a-c) Differences in the phosphorylation of CREB and expression of BDNF between groups in the PFC and hippocampus (n = 3-4). (d, e) pCREB and BDNF immunofluorescence was assessed in the PFC and hippocampus (n = 3-4). Mean ± SD. ^#^*P < *0.05 versus normal group; ^*∗*^*P* < 0.05 and ^*∗∗*^*P* < 0.01 versus control group. Scale bar = 200 *μ*m.

Previous literature has shown a correlation between depression and CREB/BDNF signaling; therefore, we examined whether FX extract affects the activity of CREB and BDNF in this animal model (Figure 4(a)). We found that there was a decrease in CREB phosphorylation and BDNF expression in the hippocampus following chronic stress and this decrease was attenuated by treatment with 100 mg·kg^−1^ FX extract or doxepin (Figure 4(b); P.C., F_(3,8)_ = 5.24, p = 0.027; Hippo, F_(3,8)_ = 7.093, p = 0.0121 and Figure 4(c); Hippo, F_(3,12)_ = 10.81, p = 0.001). However, we found no significant difference in prefrontal cortex. Immunofluorescence confirmed the differences in pCREB and BDNF, supporting the effect of FX extract on the hippocampus (Figure 4(d); Hippo, F_(3,11)_ = 7.982, p = 0.0042 and Figure 4(e); P.C., F_(3,12)_ = 10.35, p = 0.012; Hippo, F_(3,11)_ = 7.982, p = 0.0042); we found significant changes in pCREB and BDNF immunofluorescence in the dentate gyrus of hippocampus. These results suggest that treatment with 100 mg·kg^−1^ FX extract ameliorates chronic stress* via* CREB/BDNF signaling.

### 3.5. HPLC-UV Analysis of Different Components of* F. rhynchophylla* Hance Extract

**Figure 5:**
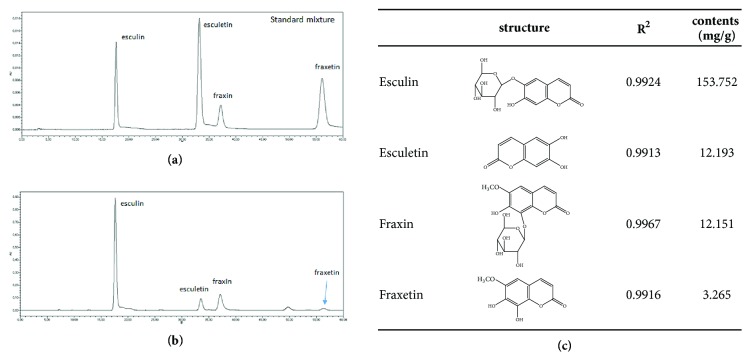
**A schematic of the experimental procedures and standardization of FX extract at 340 nm**. (a) HPLC-UV of 4 standard mixtures and (b) HPLC-UV of the FX extract. (c) Identification of the primary components from the FX extract.

The optimized HPLC-UV method was applied for the chemical profiling of FX, as shown in Figure 5. We standardized the FX extracts with esculin (153.752 mg·g^−1^), esculetin (12.193 mg·g^−1^), fraxin (12.151 mg·g^−1^), and fraxetin (3.265 mg·kg^−1^). Esculin, esculetin, fraxin, and fraxetin appeared at retention times of 17.66 min, 33.52 min, 37.29 min, and 56.55 min, respectively. Among these compounds, esculin and esculetin were detected as the major compounds of FX.

### 3.6. Effect of Different Components of* F. rhynchophylla* Hance Extract on Stress-Induced Depression

**Figure 6:**
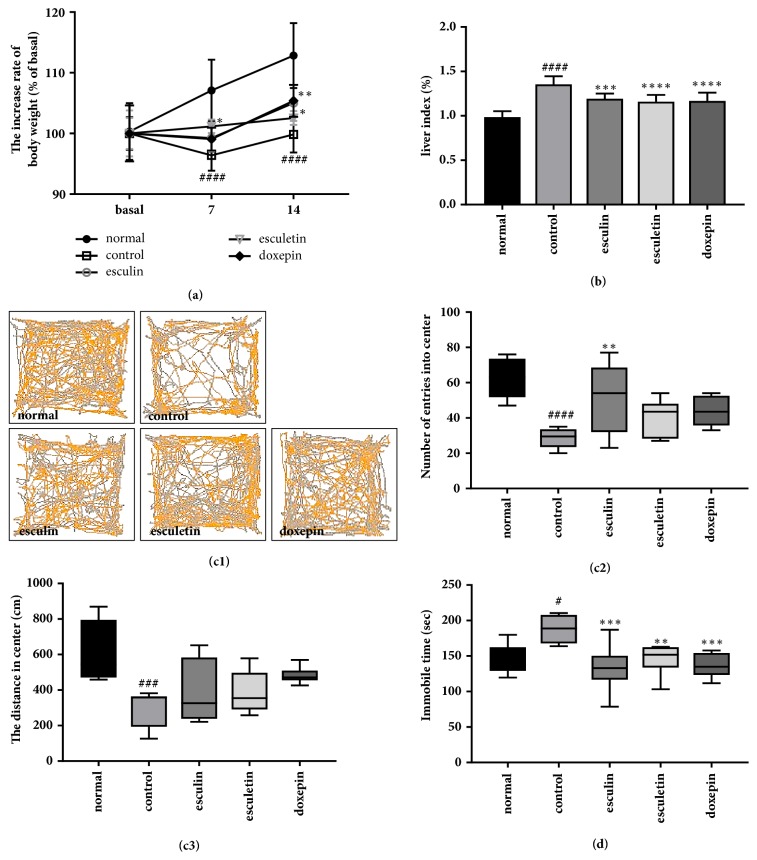
**Effect of different components of FX extract on body and relative liver weight, and depressive-like behavior**. (a) The difference in body weight between groups through the experimental procedure (n = 8-9). (b) The difference in relative liver weight between groups (n = 8-10). (c) Representative recordings of total distance travelled in OFT in all groups. Differences between the total distances travelled (n = 7-8) and mean numbers of entries into center (n = 8) in all groups. (d) Mean immobility time between groups in the FST (n = 7-8). Mean ± SD. ^#^*P* < 0.05, ^###^*P* < 0.001, and ^####^*P* < 0.0001 versus normal group; ^*∗*^*P* < 0.05, ^*∗∗*^*P* < 0.01, ^*∗∗∗*^*P* < 0.001, and ^*∗∗∗∗*^*P* < 0.0001 versus control group.

**Figure 7:**
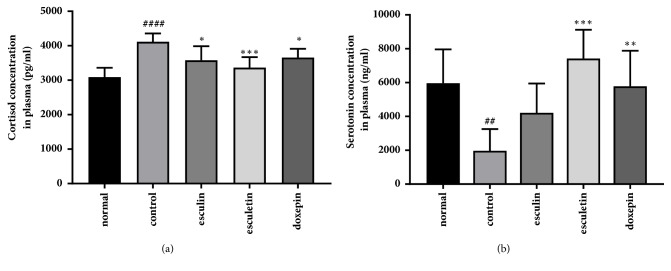
**Effect of different components in FX extract on serum cortisol and serotonin concentration**. The concentration of serum ((a), n = 7-8) cortisol and ((b), n = 6) serotonin in all groups. Mean ± SD. ^##^*P* < 0.01 and ^####^*P* < 0.0001 versus normal group; ^*∗*^*P* < 0.05, ^*∗∗*^*P* < 0.01, and ^*∗∗∗*^*P* < 0.001 versus control group.

We sought to further assess the effects of specific components of FX extract; therefore we measured depressive-like behavior, and the concentration of serotonin and cortisol following treatment with esculin, esculetin, and doxepin. The chronic stress-induced reduction in body weight was significantly ameliorated by treatment with all two components (Figure 6(a); F_(4,116)_ = 17.65, p < 0.0001). Stress can increase the relative weight of the liver. Chronic stress significantly increased the relative liver weight (% body weight) when compared with the normal group. This increase was significantly attenuated by treatment with esculin, esculetin, or doxepin (Figure 6(b); F_(4,43)_ = 24.2, p < 0.0001). Treatment with esculin significantly increased the number of entries to center in the OFT (F_(4,35)_ = 8.738, p < 0.0001); however, there was no significant difference in the distance travelled in the center (Figure 6(c)). Treatment with esculin, esculetin, and doxepin significantly attenuated the immobility time of mice in the FST (Figure 6(d); F_(4,32)_ = 7.531, p = 0.0002). Following chronic stress, the increased levels of cortisol in the plasma were significantly attenuated using esculin, esculetin, or doxepin treatment (Figure 7(a); F_(4,25)_ = 8.096, p = 0.0002). In addition, the concentration of plasma serotonin was significantly increased by treatment with esculetin or doxepin when compared with the control group (Figure 7(b); F_(4,34)_ = 13.03, p < 0.0001). These results suggest that treatments with extracted major components of FX are also effective in reducing chronic stress-induced behavioral and biochemical changes. In addition, these data suggest FX could be a potential antidepressant.

## 4. Discussion

The present study aimed to evaluate the effects of FX extract in a chronic stress-induced depression mouse model* via* electric foot shock and restraint. Treatment with FX extract prevented the chronic stress-induced body and relative liver weight loss and attenuated anxiety and despair behaviors. In addition, the concentration of serum serotonin and cortisol returned to normal levels following by FX extract treatment. These data suggest that FX extract has antidepressant properties. Furthermore, we showed that it exerts these effects by modulating the secretion of proinflammatory cytokines and CREB/BDNF signaling in the prefrontal cortex and hippocampus.

Chronic stress-induced depression was induced in mice by sustained restraint and electric foot shock for 2 wks. Long-term restraint stress adversely affects the hypothalamic–pituitary–adrenal (HPA) axis, leading to symptoms of depression, such as anxiety, despair, and cognitive impairment, in animals [39]. In addition, electric foot shock is a complex stressor, inducing physical and emotional factors that cause behaviors that reflect human depression and anxiety [40]. Our study suggests that FX is a therapeutic natural product that can improve depressive-like behavior due to these stresses.

Cortisol is secreted from the adrenal cortex into the circulation in response to stress. High concentrations in plasma cortisol of humans and mice have been reported in acute or chronic stress [41, 42]. Serotonin contributes to the feeling of happiness and can be found in the gastrointestinal tract, platelets, and central nervous system of animals and humans [43]. Depression impairs the activity of monoamine metabolites, such as serotonin and noradrenaline, in plasma and cerebrospinal fluid [44]. This study has shown that plasma cortisol and serotonin returned to normal levels following treatment with FX extract in chronic stress-induced mice.

Psychological stress increases IL-6 and its receptors* via* NF-*κ*B activation in the brain [45]. Chronic stress paradigms increase IL-12 levels in basal plasma [46]. Other studies have reported elevated proinflammatory cytokines, such as IL-1*β*, IL-6, IL-8, IL-12, and TNF*α*, in the serum of depressed patients [21, 22]. Treatment with FX extract reduced the expression of* NF-κB*,* IL-6,* and* IL-12* mRNA in prefrontal cortex and hippocampus, which were increased by chronic stress. These results suggest that FX extract may modulate the NF-*κ*B mediated proinflammatory cascade associated with chronic stress-induced depression.

Changes in BDNF have been associated with anxiety-related disorders and major depressive disorder [47]. Reductions in hippocampal BDNF are found in patients with major depression, which may affect neurogenesis. Furthermore, the concentration of BDNF is increased following antidepressant treatment and known to promote hippocampal neuronal survival and activity [48]. Chronic unpredictable stress is associated with depression and with learning and memory impairments* via* the cAMP/protein kinase A (PKA)/CREB/BDNF signal cascade [49]. In our study, CREB phosphorylation and BDNF expression were reduced following chronic stress. Treatment with FX extract significantly ameliorated this effect, suggesting that it may affect chronic stress* via* the CREB/BDNF signaling pathway. Moreover, we found these signaling changes in the dentate gyrus, which plays a functional role in stress and depression [50].

The major components of FX that have been shown to be effective in chronic stress-induced depression are esculin and esculetin. Esculetin, a component of FX, improves LPS-induced depressive-like behavior. Esculetin ameliorates anxiety and depressive-like behavior by modulating hippocampal BDNF/tropomyosin receptor kinase B (TrkB) signaling, neuroinflammation, and oxidative stress [51, 52]. In our study, both esculin and esculetin alleviated the chronic stress-induced increase in relative liver weight and decrease in despair-associated behavior and cortisol concentration. Additionally, esculin reduced anxiety-associated behaviors. Conversely, only esculetin significantly affected serum serotonin concentration. In summary, we have identified specific FX component-associated effects, which will help clarify the role of these compounds in future antidepressant treatment.

Several studies have assessed the interaction between inflammatory factors, stress hormones, and depressive-like symptoms. The chronic stress paradigm has been reported to increase the concentration of both IL-12 and stress hormones [46, 53]. Other studies have suggested that proinflammatory cytokines release glucocorticoids that stimulate the HPA axis and inhibit neurogenesis [23]. In addition, stress hormones, in combination with major depressive disorder, reduce BDNF in the hippocampus and are involved in reduced neuronal survival [54]. These studies are associated with the interaction of monoamines, inflammation, and neurogenesis in stress and depression. Further studies are necessary to assess the potential neuroendocrine mechanisms of FX extract that mediate chronic stress.

## 5. Conclusion

In summary, two weeks of chronic stress induced anxiety and despair in mice, as measured by the OFT and FST and by levels of cortisol, serotonin, proinflammatory cytokines, and CREB/BDNF. However, FX extract effectively ameliorated the depressive behaviors and restored neurotransmitter and hormone secretion to normal levels. Furthermore, FX extract also modulated proinflammatory cytokine and CREB/BDNF pathways. In conclusion, FX extract may be a potent option for long-term treatment against chronic stress-induced depression by controlling inflammation and neurogenesis-related signaling.

## Data Availability

The datasets used and/or analyzed in the current study are available from the corresponding author upon reasonable request. The role of the funding body in the design of the study and collection, analysis, and interpretation of data and in writing the article should be declared in this request.
