# Corrigendum to “Antidepressant Effect of* Fraxinus rhynchophylla* Hance Extract in a Mouse Model of Chronic Stress-Induced Depression”

**DOI:** 10.1155/2019/4672059

**Published:** 2019-05-28

**Authors:** Yu Ri Kim, Bo-Kyung Park, Young Hwa Kim, Insop Shim, In-Cheol Kang, Mi Young Lee

**Affiliations:** ^1^Clinical Medicine Division, Korea Institute of Oriental Medicine, Daejeon 34054, Republic of Korea; ^2^Department of Physiology, School of Medicine, Kyung Hee University, 1 Kyungheedae-ro, Dongdaemun-gu, Seoul 02454, Republic of Korea; ^3^Department of Biological Science, College of Life and Health Sciences, Hoseo University, Asan 31499, Republic of Korea

In the article titled “Antidepressant Effect of* Fraxinus rhynchophylla* Hance Extract in a Mouse Model of Chronic Stress-Induced Depression” [1], an incorrect version of Figure 1(c1) was published due to a mistake made by the authors while editing the figure. The corrected figure is shown below.

## Figures and Tables

**Figure 1:**
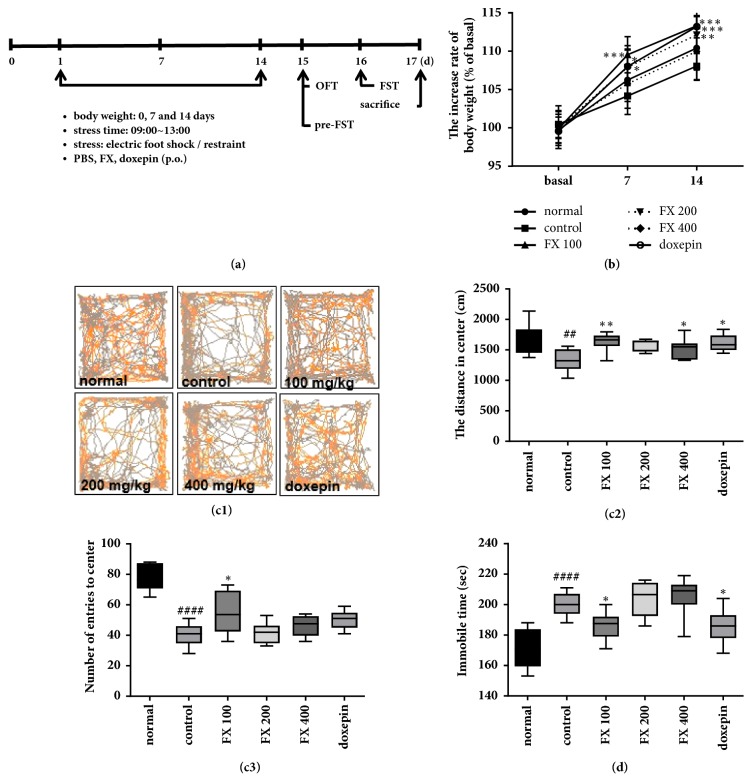
**Effect of FX extract on body weight and depressive-like behavior**. (a) Experimental procedure schematic. (b) Body weight in all mice groups as a % of baseline body weight (n = 8-10). (c1) Representative recordings of total distance travelled in Open Field Test in all groups. (c2) Differences between the total distances travelled in center (n = 8-9). (c3) Differences between mean numbers of entries into center in all groups (n = 8). (d) Mean immobility time between groups in the Forced Swim Test (n = 11). Mean ± SD. ^##^*P* < 0.01 and ^####^*P* < 0.0001 versus normal group; ^*∗*^*P* < 0.05, ^*∗∗*^*P* < 0.01, and ^*∗∗∗*^*P* < 0.001 versus control group.
